# Fault Diagnosis of Active Magnetic Bearing–Rotor System via Vibration Images

**DOI:** 10.3390/s19020244

**Published:** 2019-01-10

**Authors:** Xunshi Yan, Zhe Sun, Jingjing Zhao, Zhengang Shi, Chen-An Zhang

**Affiliations:** 1 Institute of Nuclear and New Energy Technology, Tsinghua University, Beijing 100084, China; sun_zhe@mail.tsinghua.edu.cn (Z.S.); zhao-jj@tsinghua.edu.cn (J.Z.); shizg@tsinghua.edu.cn (Z.S.); 2 Key Laboratory of Advanced Reactor Engineering and Safety, Ministry of Education, Beijing 100084, China; 3 Collaborative Innovation Center of Advanced Nuclear Energy Technology, Beijing 100084, China; 4 State Key Laboratory of High Temperature Gas Dynamics, Institute of Mechanics, Chinese Academy of Sciences, Beijing 100190, China

**Keywords:** fault diagnosis, vibration signals, active magnetic bearing, rotary machinery, AdaBoost

## Abstract

As important sources in fault diagnosis of rotary machinery, vibration signals are usually processed in the time or frequency domain as features to distinguish different classes of faults. However, these kinds of processing methods always ignore the corresponding relations among multiple signals, resulting in information loss. In this paper, a new fault description strategy named vibration image is proposed, based on which three new kinds of features are extracted, containing coupling information between different channels of vibration signals. Additionally, a new feature fusion method called two-layer AdaBoost is designed to train the fault recognition model, which avoids overfitting when the dataset is not large enough. Features based on vibration images combined with two-layer AdaBoost are adopted to diagnose faults of rotary machinery. Taking an active magnetic bearing-rotor system as the experimental platform, a dataset with four classes of faults is collected and our algorithm achieves good performance. Meanwhile, features based on vibration images and two-layer AdaBoost are both proved to be efficient separately.

## 1. Introduction

Rotary machinery plays an irreplaceable role in modern industry. Over the past decades, the safety of equipment has received more and more attention and the fault diagnosis of rotary machinery has become a hot research topic. Faults not only include the imbalance of the rotor itself but also may occur at the bearings, gear boxes and couplings, which determines the variation and complexity of faults. Hence, how to describe faults is key to fault diagnosis. Various sources such as vibration [1], electric current and acoustic signals [2,3] are used in diagnosis. Generally, vibration signals are important sources of faults and contain abundant information about running states of rotary machinery, which are widely used to extracted features in fault description. Most researchers in this field have devoted their energy to research vibration signals and proposed a large number of methods [4,5].

As a kind of time series, vibration signals are generally processed via time or frequency domain analyses, which are the mainstream methods. The tools widely used in the signal processing and communication field are also used to describe faults of rotary machinery. These methods include Fourier transform, statistical moments [6], autoregressive [7], Walsh transform [8], power spectral density [9], and entropy [10]. They can be easily calculated, but have limited ability to describe faults. To date, these simple features are still used in many algorithms as complementary features or one of multiple features [11], and many complex features are designed based on them.

Due to the non-stationary and nonlinear characteristics of vibration signals, time–frequency methods are introduced into fault signal analysis. Short time Fourier transform applies a time variable to the traditional Fourier spectrum, and moves a short time window along time to divide a signal into slices, which reflects the time–frequency characteristic of the vibration signal in the short time period [12,13]. Furthermore, wavelet transform achieves multi-resolution partitioning in frequency and time, thereby achieving a more detailed observation of the fault signal in two dimensions [14]. Empirical mode decomposition (EMD) is carried out to decompose the vibration signals into intrinsic mode functions (IMFs), which are always processed into more descriptive features, and it is applicable to non-stationary and nonlinear vibration signals [15].

In recent years, with the advancement of machine learning, fault diagnosis technology has been greatly promoted. Fault signals are projected into the nonlinear space for processing, according to locally linear embedding [16], sparse representation [17] or auto-encoder [18], to obtain more distinctive feature representation. Convolutional neural network is introduced with raw signals to achieve perfect accuracy without learning features manually [19]. Recurrent neural network with auto-encoder [20] is used to deal with multiple vibration time sequences.

Most fault diagnosis algorithms excavate the time–frequency characteristics from vibration signals and describe the faults of rotary machinery. However, vibration signals are collected from different sensors and processed equally. The relationship between vibration signals collected from sensors fixed in different locations is always ignored, although this factor is important to distinguish various faults. For example, as the vibration signals collected from sensors fixed at the same axis line are coupled, when an impulse is added to the location of one sensor, it will also obtain the response from another sensor at the axis line. Hence, there exists a coupling relationship between the vibration signals from different sensors. If these vibration signals are processed separately and the coupling relationship is ignored, it will lead to information loss.

Shaft orbit [21,22] is one kind of method considering the relationship of different sensors, some specific shapes of which correspond to faults. However, this method is limited to describing the signals from sensors in one radial plane. A new fault description called vibration image is proposed in this paper. In this approach, vibration image is synthesized by any two channels of vibration signals. The coupling of vibration signals at different positions is described by extracting the features of vibration images. Through a new fusion algorithm, two-layer AdaBoost, the recognition results of several different feature operators are synthesized to realize the final fault diagnosis.

**Figure 1:**
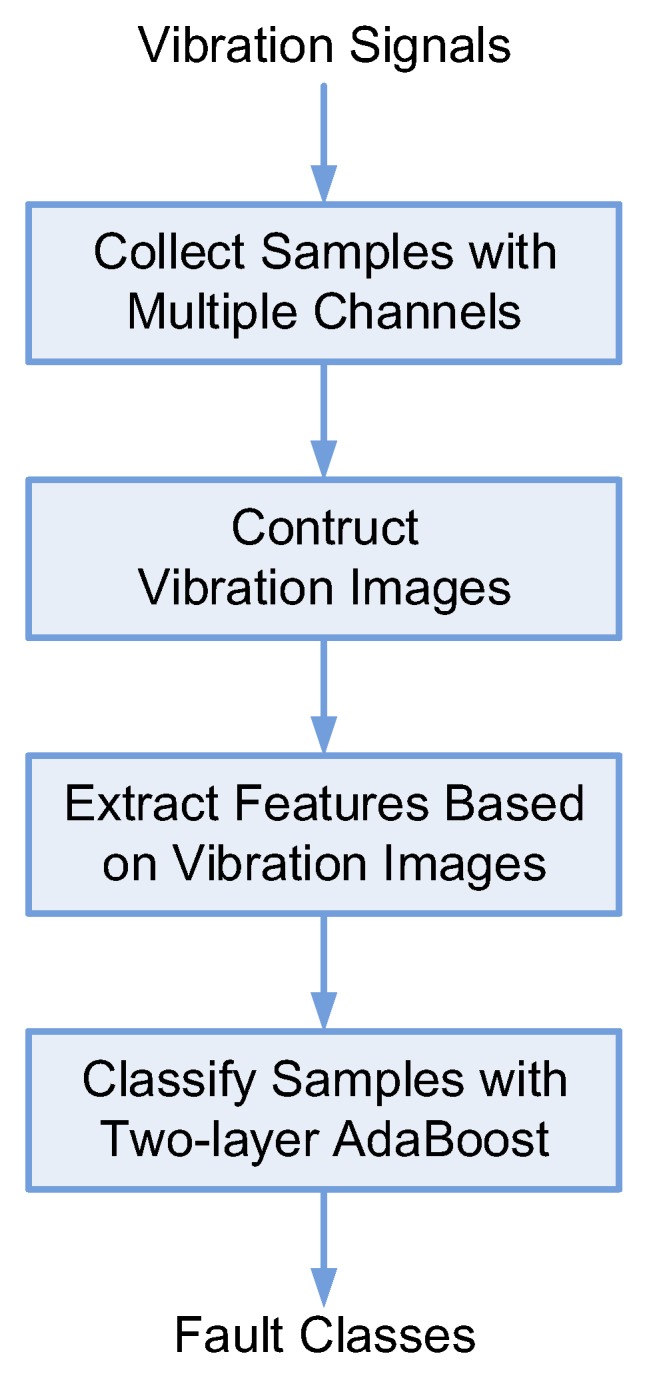
Flowchart of the proposed algorithm.

As a research target, rotating machinery supported by active magnetic bearing (AMB) is introduced and the above algorithm is designed in combination with the characteristics of the active magnetic bearing-rotor system. The corresponding research results can also be extended to the fault diagnosis of other rotary machinery. Figure 1 gives the flowchart of the proposed algorithm.

The main contributions of this paper can be summarized as follows.

(1) A new fault description strategy termed vibration image is proposed to describe faults in rotary machinery, to capture the coupling information between different sensors installed in various positions.
(2) Three new features based on vibration image are created to describe faults in different views.
(3) To overcome the problem of overfitting, a new fusion algorithm called two-layer AdaBoost is employed to fuse multiple features and diagnose faults in rotary machinery.

In this manuscript, AMB is introduced in Section 2. Section 3 details the concept of vibration images and provides a description of three kinds of features based on vibration images. The classification algorithm AdaBoost is given in Section 4, and the fusion strategy two-layer AdaBoost is detailed. In Section 5, a series of experiments are presented to prove the effectiveness of the proposed algorithm. Section 6 summarizes the full text and highlights the future research directions.

## 2. Active Magnetic Bearing

An AMB–rotor system is a typical mechatronics rotary machinery. Compared to mechanical bearings, AMB does not need lubrication. Moreover, it can reduce friction and increase efficiency because it does not contact the rotor. AMB can also reduce the excessive vibration of a high-speed rotor system at the critical speed, which helps the motor run over the critical speed stably. These remarkable advantages enable the application of AMB to many fields, such as high speed motors, centrifugal compressors, high-speed flywheels for storage energy, molecular pumps and turbines [23,24,25].

**Figure 2:**
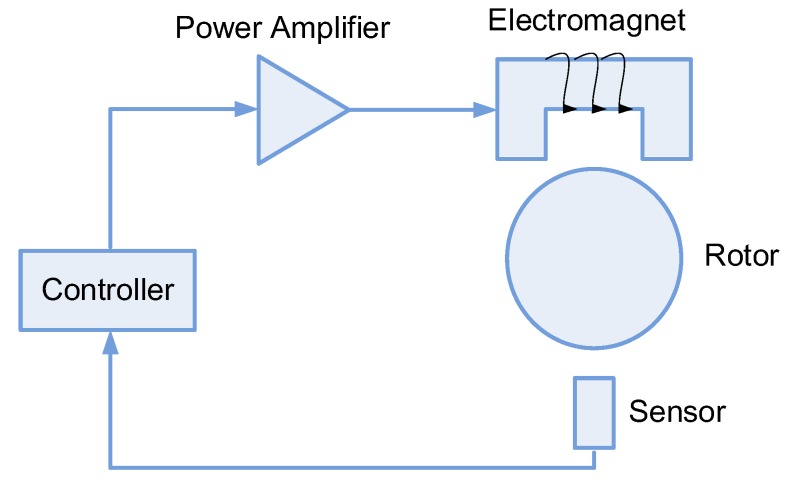
The basic model of AMB.

An AMB–rotor system is a closed-loop control system consisting of a rotor, displacement sensors, a controller and a power amplifier. Figure 2 shows a basic model of an AMB system. The basic principle is that, when the rotor veers off center position, the displacement sensors measure and send the deviation to the controller. The controller calculates and drives the power amplifier to change the current of the coils, thereby changing the electromagnetic force to drive the rotor back to the center position. This is a dynamic procedure. Due to precise active control of AMB, the vibration can be limited to a small range under normal working conditions, whether there is an increase in speed or a load change.

**Figure 3:**
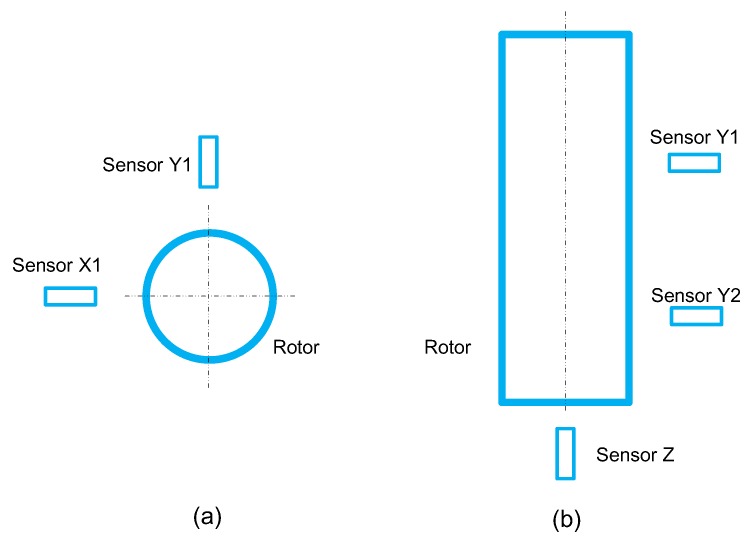
Placement of displacement sensors in an AMB–rotor system. (**a**) shows sensors arranged in one radial plane and (**b**) is a sensor arrangement distributed along the axis.

The displacement sensor of an AMB–rotor system measures the offset of the rotor from the center position. This can not only be utilized as feedback to ensure the stability of the system but is also viewed as the vibration of the rotor. Therefore, an AMB–rotor system has a natural advantage in fault diagnosis. It is not necessary to add new vibration sensors and the existing displacement sensors can be used for vibration analysis to achieve the target of fault diagnosis. Figure 3 shows the placement of sensors in an AMB–rotor system, and an apparent coupling relationship is shown to exist between the vibration signals from the five displacement sensors. Hence, there is a need for the design of a kind of feature to describe the relationship between different channels of vibration signals.

## 3. Vibration Image

Fault diagnosis is essentially a pattern recognition problem, and feature extraction is the key to pattern recognition. Hence, the method chosen to describe the faults effectively decides the accuracy of the fault diagnosis. The vibration image approach and three features based on it are detailed in the following paragraphs.

### 3.1. Basic Form of Vibration Image

**Figure 4:**
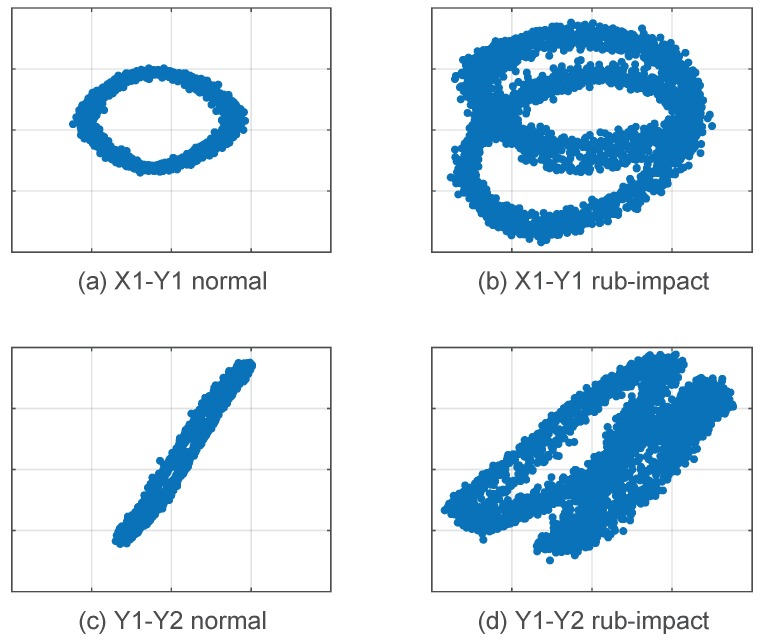
Typical vibration images created by different channel combinations.

Multiple vibration signals collected from different sensors are processed separately, and the coupling relationship between them is not considered. As previously mentioned, the correlation exists and the information is useful to distinguish different classes of faults. Inspired by orbit shaft, two channels of vibration signals from different sensors are laid together, and pairs of vibration signals at the same time are viewed as point couples in orthogonal coordinates. All the point couples form a trajectory and two channels represent the vertical and horizontal coordinates, separately. We call this trajectory in the coordinates vibration image and refer to the point couples as vibration points. When the two channels of signals are two orthogonal directions in the same radial plane, the vibration image is equivalent to the orbit shaft. The vibration image method generalizes the concept of orbit shaft, and extends the signals to any two channels, which provides more chances to create new features to explore the relationship between vibration signals. Figure 4 shows typical vibration images created by various channel combinations. It is obvious that vibration image is different from the traditional shaft orbit method, which is approximately circular in a normal state while vibration image presents with various shapes.

In the traditional shaft orbit method, the two channels of signals should be captured by two orthogonally placed sensors in the same radial plane. When the machine runs in its normal state, the shaft orbit is a circle or an approximate circle. The vibration image approach relaxes the requirements of the positions of the sensors. As long as the relative positions of the two sensors are fixed, the two vibration signals captured by them can construct a vibration image. When the system is in a normal state, the vibration image is a certain shape. When the system is in a fault state, the vibration image seriously deviates from the standard shape. Taking the Y1-Y2 signal combination in Figure 4 as an example, the vibration image in normal state is an inclined line segment (see Figure 4c). When it is in the rub-impact state, the line segment deforms into another shape (see Figure 4d). Vibration images exhibit a difference between normal and abnormal states, which ensures that vibration images can use two arbitrary channels of signals to diagnose faults.

When there are more than two channels of signals, the vibration image is constructed by arbitrarily selecting two channels; hence, multiple vibration images can be generated. For example, an AMB–rotor system with five sensors can construct 10 vibration images. Features are extracted from each vibration image. Multiple types of features from vibration images are combined by two-layer AdaBoost. This feature construction mechanism provides plenty of feature combinations for recognizing faults from different views, which was used in the experiments.

The basic idea of vibration image is intuitive and simple, and has many advantages. First, vibration image converts one-dimensional (1D) signals into two-dimensional (2D) signal combination. In fact, an image is a 2D signal, and all image processing methods are also considered signal processing methods. Hence, a variety of existing image processing and description algorithms can be used to analyze vibration images. Second, the vibration image reflects the coupling relationship between the two channels of signals. The amplitude and phase of two channels of signals are shown in the vibration image and the coupling relationship between them is also presented in such an intuitive form, which was previously hidden in a separate signal and difficult to express. Third, a vibration image can be constructed by selecting two channels of vibration signals arbitrarily. This feature construction mechanism provides plenty of feature combinations for recognizing faults from different views.

Similar to the shaft orbit method, vibration image is an identical description of faults and requires basic features to be described. Based on vibration image, we design three description methods from views of vibration amplitude, vibration phase, and frequency analysis.

### 3.2. Histogram of Vibration Image

The amplitude of the vibration signal is an important characteristic of faults. To describe the two channels of vibration signals and the coupling relationship between them simultaneously, a histogram of vibration image (HVI) is designed to achieve this work. HVI can be constructed in the following steps.

First, calculate the vibration image center. Assuming the vibration points as $\left(x_{i},y_{i}\right)$, i=1,2,…,M, and *M* is total number of vibration points, the center coordinate $\left(\bar{x},\bar{y}\right)$ is calculated as Equation (1).

(1) $$\begin{array}{cc} \bar{x} & =\frac{1}{M}\sum_{i}x_{i}\\ \bar{y} & =\frac{1}{M}\sum_{i}y_{i} \end{array}$$

Second, remove the unexpected outliers. Some vibration points are affected by noises and other disturbing sources, and have abnormal amplitude, which should be omitted. Set the center of $\left(\bar{x},\bar{y}\right)$ to draw a vibration circle with *R* as the radius, while the points outside the circle are regarded as noise points. Calculate the Euclidian distance $A_{i}$ between the center and all vibration points according to Equation (2)

(2) $$A_{i}=∥P\left(x_{i},y_{i}\right)-P\left(\bar{x},\bar{y}\right)∥$$

and retain the points with the distance $A_{i}<R$. The radius *R* is calculated based on Equation (3). *T* represents the ratio of the vibration points retained to all vibration points, set to 95% empirically.

(3) $$\begin{array}{cc} R & =A_{m}\\ \mathrm{subject}\;\mathrm{to}:T & =\frac{\mathrm{count}(A_{i}\leq A_{m})}{M},i=1,2,\ldots ,M \end{array}$$

Third, form the histogram feature. Divide the vibration circle into *N* rings; that is, *R* is partitioned into *N* equivalent intervals. $z_{i}$ represents *i*th point locating the $z_{i}$th ring. The *k*th bin value of the histogram is set to the number of points fallen into the *k*th ring (see Equation (4)). Finally, the histogram is normalized. The dimension of HVI is *N*.

(4) $$\begin{array}{cc} \mathrm{zero}(x) & =\left\{\begin{array}{c} 1,x=0\\ 0,x\neq 0 \end{array}\right.\\ \mathrm{hvi}(k) & =\sum_{i}\mathrm{zero}(z_{i}-k) \end{array}$$

**Figure 5:**
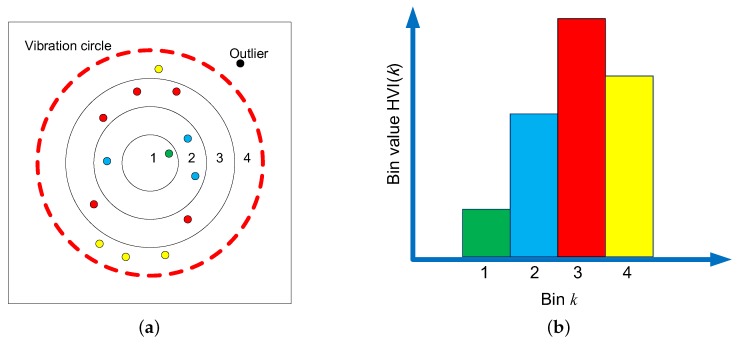
The schematic of HVI: (**a**) distribution of vibration points; and (**b**) histogram of vibration points.

HVI uses the form of a histogram to describe the distribution of vibration amplitude variation in a vibration image. Obviously, it reflects the coupling relationship between the two channels of signals, and it is also invariant to rotation of the vibration image, which means it is more robust. Figure 5 shows the schematic of HVI.

### 3.3. Histogram of Oriented Vibration Image

Different from HVI, a histogram of oriented vibration image (HOVI) represents the distribution of vibration points in various directions. HOVI is calculated according to the following procedure.

The first step is similar to that of HVI. Find the vibration center $\left(\bar{x},\bar{y}\right)$ and calculate the Euclidian distance $A_{i}$ between all points and the center. Remove the outliers when $A_{i}>R$. *R* is the radius of the vibration circle.

Second, calculate the phase of each point according to Equation (5). The phase $\theta_{i}$ represents the angle between the horizon direction and the vector connecting the vibration point with the center in the vibration image.

(5) $$\begin{array}{c} \theta_{i}=\arg\left(\left(x_{i}-\bar{x}\right)+j\left(y_{i}-\bar{y}\right)\right),\;\;\;\;\;\;\;\theta_{i}\in \left[0,2\pi \right) \end{array}$$

Third, divide the phase space into *N* intervals and find $z_{i}$ which represents where each vibration point $\left(x_{i},y_{i}\right)$ falls in (see Equation (6)). The bin value of HOVI is the average amplitude of the points falling into the corresponding phase interval (see Equation (7)).

(6) $$\begin{array}{c} z_{i}=\left\lceil \frac{\theta_{i}}{\frac{2\pi }{N}}\right\rceil \end{array}$$

(7) $$hovi\left(k\right)=\frac{\sum_{i}A_{i}\mathrm{zero}\left(z_{i}-k\right)}{\sum_{i}\mathrm{zero}(z_{i}-k)}$$

**Figure 6:**
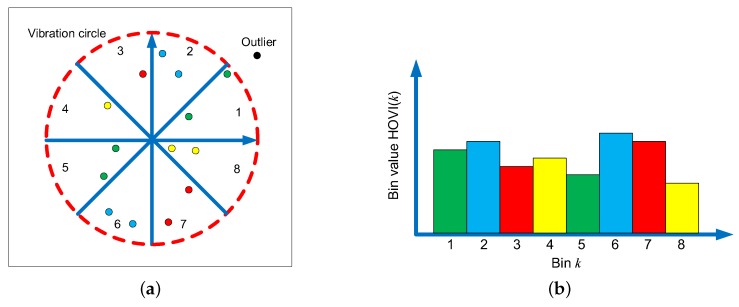
The schematic of HOVI: (**a**) distribution of vibration points in various directions; and (**b**) histogram of oriented vibration points.

HOVI is a description of vibration images used to explore the coupling relationship between two vibration signals. Different from HVI, HOVI focuses on using the phase of vibration points to describe the distribution of points in different directions. *N* is the dimension of HOVI. As *N* increases, the division of the vibration image plane becomes finer and the description of the vibration image becomes more accurate. Figure 6 shows the schematic of HOVI.

### 3.4. 2D Fast Fourier Transform of Vibration Image

In the perspective of time analysis, HVI and HOVI represent the distribution of the vibration amplitude and phase, respectively, and can be used to reveal the coupling relationship of vibration signals. Learning from time–frequency analysis methods, vibration image can also draw more meaningful conclusions in the frequency domain. Fourier analysis is an important frequency analysis tool, and vibration images can be seen as a two-dimensional image signal; hence, 2D fast Fourier transform (2DFFT) is selected to describe the frequency characteristic of the vibration image. The calculation procedure of vibration image 2DFFT is detailed as follows.

First, divide the two coordinates of the vibration image into N equal intervals, so that the entire vibration image is partitioned into $N^{2}$ grids. Each grid can be considered as a “pixel” of vibration image. If vibration points fall into the grid, we set the value of the grid as 1, otherwise 0. The vibration image transforms into 2D 1–0 matrix.

Second, apply 2D fast Fourier transform to the 2D 1–0 matrix and obtain the frequency amplitude and phase map that have same size as the 2D 1–0 matrix.

Third, recognize the amplitude and phase map as 1-D vectors and combine them together as a feature vector. The dimension of the 2DFFT feature is $2N^{2}$.

**Figure 7:**
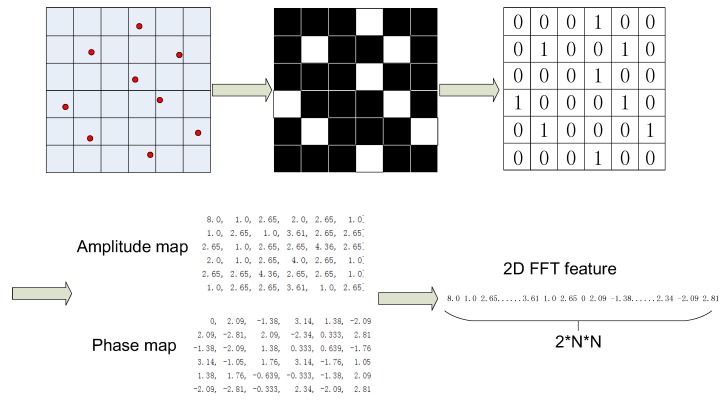
Explanation of 2DFFT.

Three types of features based on vibration image describe the vibration signals from different perspectives. Different from HVI and HOVI described above, 2DFFT discovers coupling information of two channels of vibration signals in the frequency domain. 2DFFT includes amplitude and phase of FFT result of vibration image and gives much more discriminative information to distinguish the faults of rotary machinery. Figure 7 shows the calculation procedure of 2DFFT.

### 3.5. Summary

Three features based on the vibration images are used to characterize faults. They are viewed, respectively, from the amplitude distribution of vibration points synthesized by two channels of vibration signals, the distribution of vibration points in different directions, and the analysis of vibration points in the frequency domain. The high-level feature description of the vibration image is formed by these three types of features.

## 4. Classification Algorithm

Essentially, fault diagnosis of rotary machinery is a problem of pattern recognition. Besides feature extraction, classification is another important problem. Many common methods in machine learning field are brought into fault diagnosis. Support Vector Machine (SVM) [21], K-Nearest Neighbor (KNN) [26], Naive Bayesian [27], and Fisher Discrimination as well as their variants are widely used, although some other popular methods in recent years are introduced such as deep learning [28,29]. In our algorithm, we introduce AdaBoost and design its variants to solve the multiple feature fusion problem.

### 4.1. AdaBoost

AdaBoost is an ensemble classification algorithm [30,31] that is widely used in face detection, image retrieval and action recognition [32,33]. It adaptively combines a collection of weak learners to form a strong classifier by a series of weights. The weights are dynamically updated according to the errors in the previous iteration, so that a wrongly classified sample will be more likely to be correctly classified in the next iteration. The weak learners are basically demanded to perform better than random decisions. For some reasons, it seems that AdaBoost is more resistant to overfitting than the other algorithms mentioned above. The final form of AdaBoost is shown in Equation (8):

(8) $$F=\sum wH\left(x\right)$$

where *x* is a sample. *H* represents weak learner and *w* is the trained weight. *F* is the result of combining weak learners, the dimension of which is equal to the number of sample classes. Each element of *F* indicates the probability of the sample *x* belonging to the corresponding class. *D* is the sample class which is given as the final decision of classifier (see Equation (9)).

(9) $$D=\underset{j}{\arg\max}F\left(x,j\right)$$

In fact, the basic idea of AdaBoost is similar to fusion algorithms combining different types of features according to a group of weights. Hence, AdaBoost outperforms most simple classification algorithms.

### 4.2. Two-Layer AdaBoost

According to Vapnik–Chervonenkis dimension theory (VC dimension theory), the ability of a classification algorithm is related to the dimension of the sample feature and the size of the dataset. As the dimension of features is closer to the number of training samples, the generation ability of the classification algorithm is weaker. VC dimension theory tells us that a high dimension feature or a small dataset is prone to overfitting. In the traditional pattern recognition problem, the dataset usually contains thousands or even millions of samples and overfitting is not considered; hence, the dimension of features can extend to the thousands or larger. However, in rotary machinery, accidents or faults only occur occasionally and it is difficult to obtain a large dataset of fault samples. Most datasets used for research contain fewer than 2000 samples, which forces the algorithms to face the contradiction between a high dimension description and few samples.

Multiple features can be used to describe faults comprehensively, but they inevitably face the high dimension problem. Thus, there is a need to design a fusion algorithm that not only fuses more information from features but also overcomes overfitting.

There exist three main strategies to fuse multiple types of features.

First, combine features directly (CFD) to describe faults. This is a simple strategy; however, it faces overfitting and is only suitable for low dimension features.

Second, adopt dimension reduction to shrink the dimensions of features. This method projects the features into another orthogonal space, and loses the residual which may include redundant descriptive information.

Third, train classifiers separately by different types of features and combine the classifiers according to a series of weights. Many methods have been developed to identify proper weights for this purpose.

In this paper, our two-layer AdaBoost algorithm is derived from the above strategies and it is designed to avoid overfitting as well as make full use of feature information. The proposal contains two layers of AdaBoost. The feature extracted from one sample is defined as $X_{i}$ (see Equation (10)):

(10) $$X_{i}=\left[X_{i1},X_{i2},\ldots ,X_{ij},\ldots ,X_{in}\right],i=1,2,\ldots ,M,X_{ij}\in R^{k_{j}}$$

where $X_{ij}$ denotes the *j*th type of feature and is also a vector with dimension $k_{j}$. *n* is the number of feature types and *M* is total number of samples.

The first layer consists of *n* AdaBoost classifiers $\left\{F_{1},F_{2},\ldots ,F_{n}\right\}$. For $F_{j}$, the training set is the *j*th type of feature $X_{ij},i=1,2,\ldots ,M$. The basic AdaBoost algorithm is used as classifier. The output $F_{j}\left(x\right)$ is an *m*-dimension vector, each element of which indicates the probability that the sample $X_{i}$ belongs to the corresponding class. *m* is the number of fault classes. $F_{j}\left(X_{ij}\right)$ can be regarded as the dimension reduction result of the primary feature vector $X_{ij}$, and drops the residual information according to AdaBoost, which ensures that the remaining information is the most discriminative.

As mentioned above, the essence of AdaBoost is to map a high dimension feature vector into an *m*-element vector with the dimension equal to the number of classes. Each element in the mapped vector corresponds to a class, and the sample belongs to the corresponding class of the largest value in the mapped vector. Hence, the element value in the mapped vector represents the probability that the sample belongs to the corresponding class. When the difference between the element values in the mapped vector is relatively small, that is, the probabilities that the sample belongs to multiple classes are similar, the method of taking the maximum element value as the result can easily cause misidentification. In the conventional methods, when various types of features pass the first layer of AdaBoost, voting or weighting is performed to combine the maximized results of the mapped vectors and much information contained in the mapped vectors is ignored. To discover more useful information, we introduce the second layer of AdaBoost instead of taking a maximum step after the first layer of AdaBoost directly.

In the second layer, all $F_{1}\left(X_{i1}\right),F_{2}\left(X_{i2}\right),\ldots ,F_{n}\left(X_{in}\right)$ are concatenated into ${\bar{X}}_{i}$ (see Equation (11)):

(11) $${\bar{X}}_{i}=\left\{F_{1}\left(X_{i1}\right),F_{2}\left(X_{i2}\right),\ldots ,F_{n}\left(X_{in}\right)\right\},{\bar{X}}_{i}\in R^{mn}$$

where ${\bar{X}}_{i}$ is the input feature vector and the dimension is m×n. The second layer of the AdaBoost classifier $\bar{F}$ is trained and the maximum element of the output $\bar{F}\left({\bar{X}}_{i}\right)$ corresponds to the class of sample $X_{i}$ (see Equation (12)):

(12) $$y_{i}=\underset{j}{\arg\max}\bar{F}\left({\bar{X}}_{i}^{\prime },j\right)$$

When a new sample ${X^{\prime }}_{i}$ comes, it is put into the first layer AdaBoost, and the output ${\bar{X}}_{i}^{\prime }=[F_{1}\left({X^{\prime }}_{i1}\right),F_{2}\left({X^{\prime }}_{i2}\right),\ldots ,F_{n}\left({X^{\prime }}_{in}\right)$ is obtained. Then, ${\bar{X}}_{i}^{\prime }$ is input into the second layer AdaBoost to obtain $\bar{F}\left({\bar{X}}_{i}^{\prime }\right)$. The recognition result ${y^{\prime }}_{i}$ is the class corresponding to maximum element of $\bar{F}\left({\bar{X}}_{i}^{\prime }\right)$.

In two-layer AdaBoost, the first layer is a dimension reduction step, which in essence shrinks multiple high dimension features into a short feature vector with the dimension of m×n, and generates a series of AdaBoost classifiers that are trained by different types of features separately. The strategy guarantees the prevention of long feature vectors and avoids overfitting according to VC dimension theory. Compared to traditional dimension reduction methods such as principal component analysis, which only provides results based on linear transformation to remove the residual, the result of the first layer extracts discriminative information and is closer to final classification result. The second layer is the fusion layer, and is combined with the output feature vector from the first layer directly similarly to CFD. AdaBoost itself weights multiple weak learners. Compared to traditional AdaBoost, the input feature of the second layer has lower dimensionality and higher discriminative ability. In summary, two-layer AdaBoost ensembles three main ideas of fusion strategies, and can achieve perfect results. The experiments prove the algorithm to be effective and efficient.

**Figure 8:**
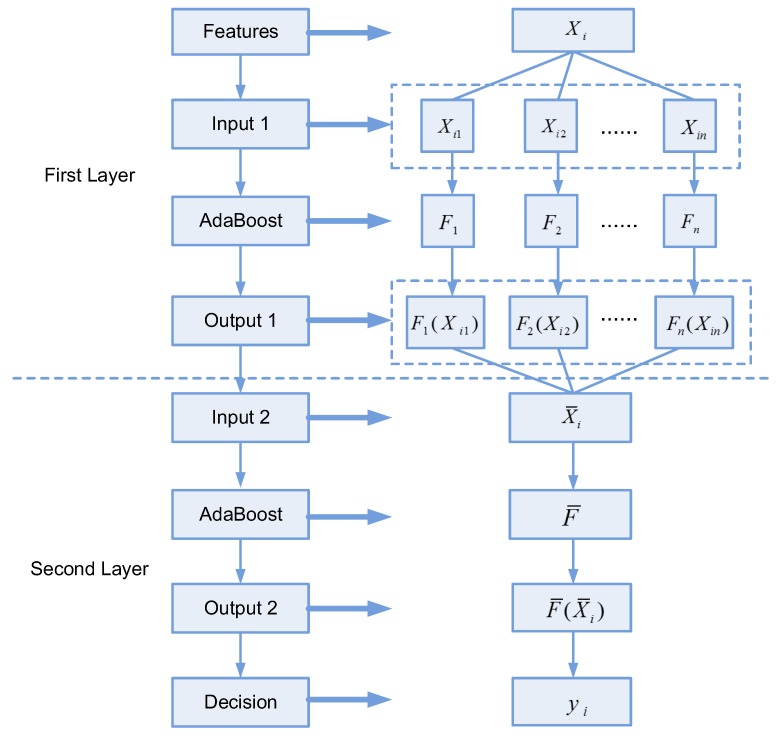
The explanation of two-layer AdaBoost.

Figure 8 gives an explanation of two-layer AdaBoost in detail.

### 4.3. The Summary of the Proposed Algorithm

**Figure 9:**
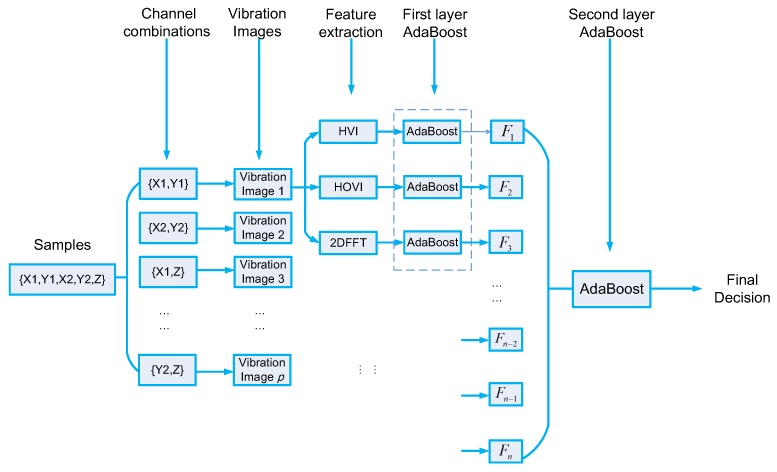
The detailed procedure of the proposed algorithm.

The algorithm procedure can be summarized in the following steps (see Figure 9).

(1) Collect the vibration signals under four conditions: normal, unbalance, misalignment, and rub-impact. Each sample consists of five channels of signals, which lay in two planes and axes named X1, Y1, X2, Y2 and Z.
(2) Select some combinations from any two of the five channels, and construct vibration images of each sample.
(3) Extract three types of features based on the vibration images for each sample.
(4) Divide the dataset into two parts, one for training and the other for testing.
(5) Via the training dataset, train the two-layer AdaBoost model.
(6) Using the two-layer AdaBoost model, test on the testing dataset and obtain the diagnosis results.

## 5. Experiments

### 5.1. Description of the Dataset and Experiment Setting

**Figure 10:**
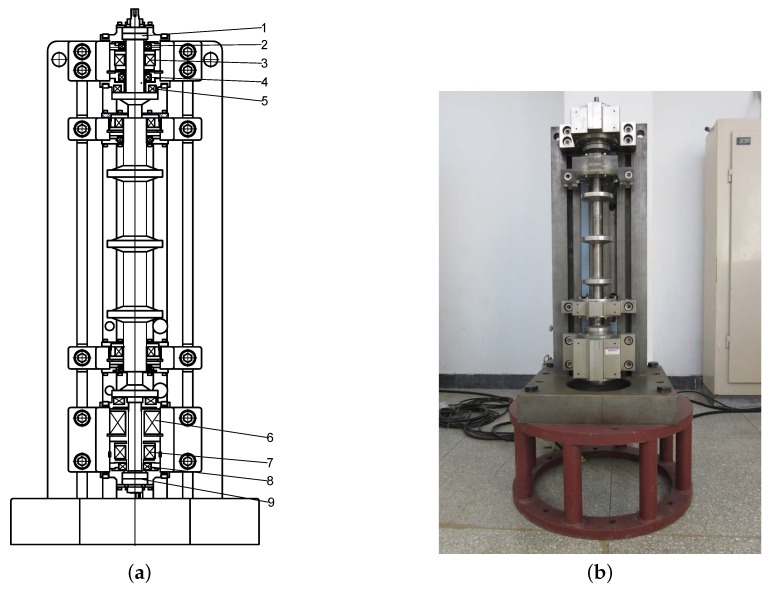
The rig of the AMB–rotor system used for experiments. (**a**) Structure: 1, upper auxiliary bearing; 2, upper radial sensors; 3, upper radial AMB; 4, axial sensor; 5, axial AMB; 6, electric motor; 7, lower radial AMB; 8, lower radial sensors; 9, lower auxiliary bearing. (**b**) Test rig.

To verify the performance of our algorithm, series of experiments were performed on a rig of an AMB–rotor system (see Figure 10). The rig included a rotor, five displacement sensors, motor, computer, and an NI signal acquisition card. The rotor was 1053 mm long and the running speed was 6000 rpm.

**Figure 11:**
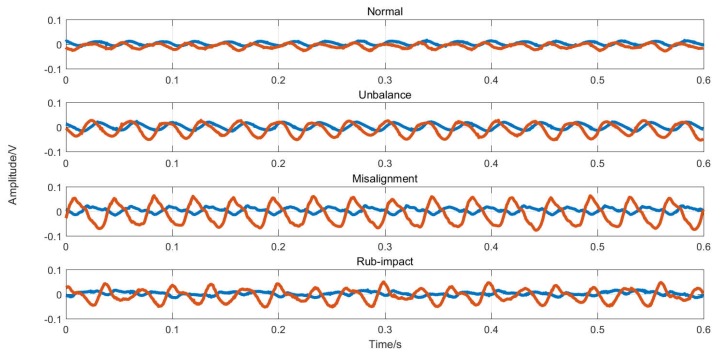
Four classes of vibration signals.

Four classes of data including unbalance, misalignment, rub-impact and normal state were collected and the size of dataset was 1445 samples. From each class, 150 samples were randomly selected as training samples and the others were utilized as testing ones (see Table 1). Each sample was denoted by a time series of vibration data consisting of five channels of signals lasting 0.6 s. The sampling rate was 25,000 points per second, and each sample consisted of 15,000 points. The time-domain waves of samples in each class are presented in Figure 11 and two of the five channels of signals are selected to show the amplitude changes over time.

All experiments were repeated 20 times by selecting training samples randomly and averaging the results to make them credible.

### 5.2. Evaluation of Three Types of Features Based on Vibration Image

Due to the difference of formation principle, we used the mark *D* to represent the dimension number of features to compare them conveniently (see Table 2). The range of *D* was from 1 to 12. There existed 10 two-channel combinations and we marked them as *C* from 1 to 10 (see Table 3). In sum, there were 360 {Type, *D*, *C*} kinds of features.

We used AdaBoost to train the three types of features separately, and found that they could achieve more than 80% accuracy at most. Although they do not satisfy the diagnosing task requirements alone, they are still powerful features. Table 4 shows the best accuracy corresponding to separate feature group {Type, *D*, *C*}. Overall, 2DFFT performs best and HVI is relatively weaker. However, these features describe vibration images from three different views. Therefore, the information contained in the features is complementary to each other and needs to be comprehensively used in the fusion algorithm.

The dimension and channel combination of the features are two factors that affect the classification performance of a single feature. Generally, with the increase of feature dimensions, the accuracy of recognition is higher, but this trend is not very clear, and, in some cases, the recognition accuracy in the case of low dimensionality is higher than that of high dimensionality.

**Figure 12:**
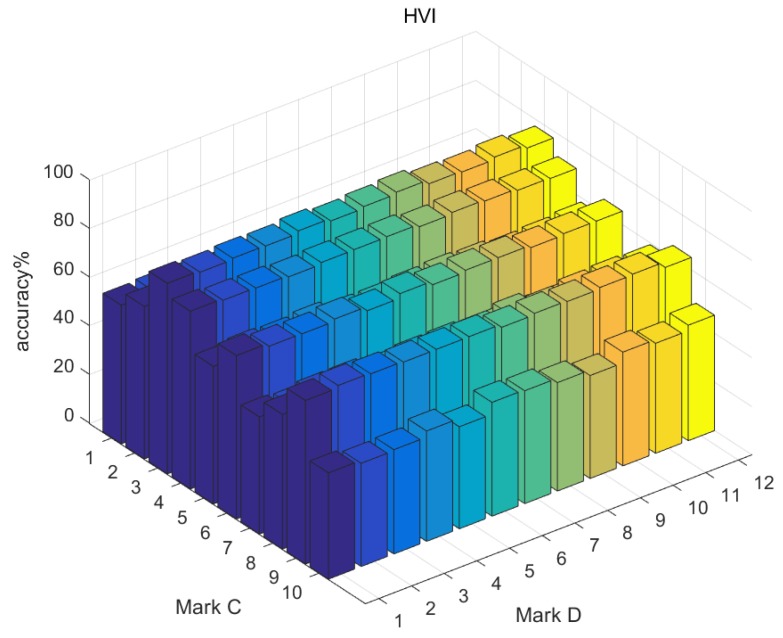
Evaluation of HVI.

**Figure 13:**
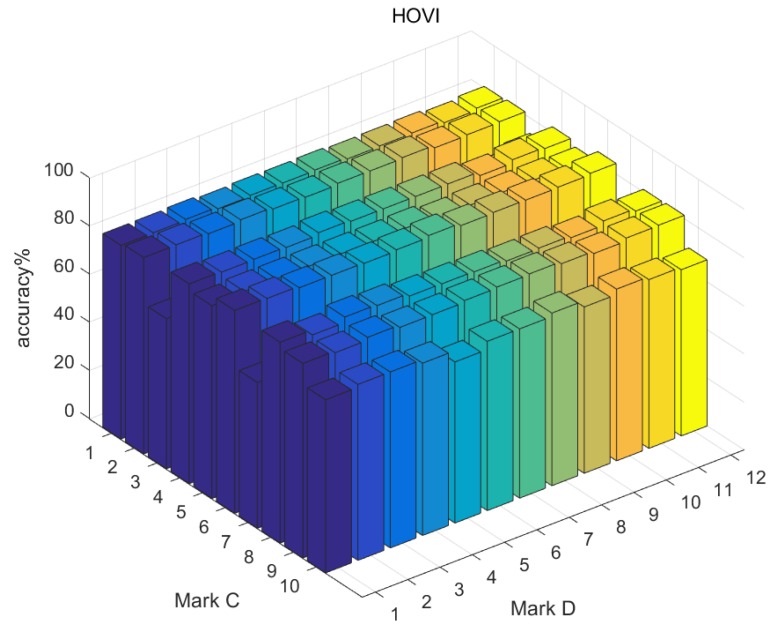
Evaluation of HOVI.

**Figure 14:**
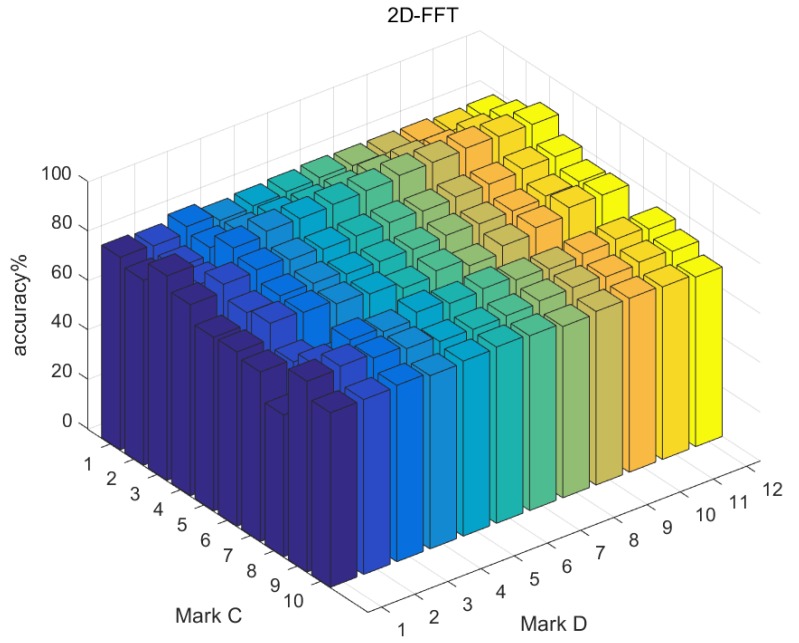
Evaluation of 2DFFT.

Compared with feature dimension, channel combination has more obvious influence on the recognition results. Figure 12, Figure 13 and Figure 14 presents recognition results of the three types of features separately, and it can be seen that, under the Y2-Z channel combination, the recognition accuracy of three types of features are all less than 70%, while, under the X1-Y2 combination, the recognition accuracy of the three types of features are all more than 70%.

**Figure 15:**
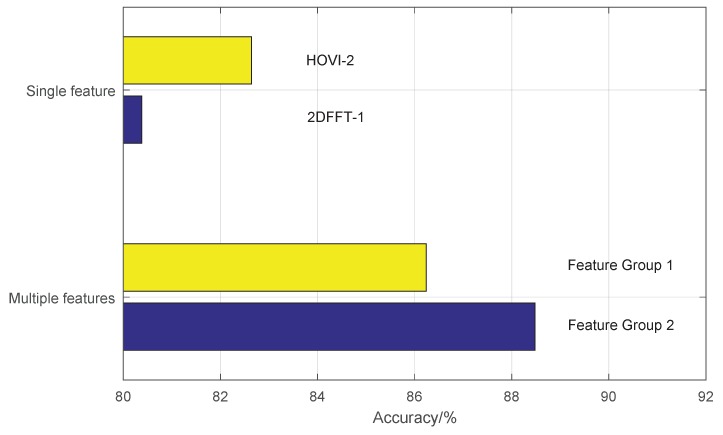
Comparison of two groups of features with two-layer AdaBoost.

Figure 15 shows the comparison results of two feature groups. Feature Group 1 includes three features including HVI-1, HOVI-1, and 2DFFT-1. In Feature Group 2, feature HOVI-2 is used instead of 2DFFT-1 (see Table 5). The recognition accuracy of individual feature HOVI-2 is higher than 2DFFT-1, but Feature Group 2 is not as good as Feature Group 1. When using a fusion algorithm, multiple kinds of features are required, and the effect of selecting several kinds of features with the best performance is not necessarily optimal, because the information between several best kinds of features overlap. They are regarded as being equivalent, but the characteristics of several types with different performance may complement each other and achieve a better result.

### 5.3. Comparison with Standard Algorithms

**Figure 16:**
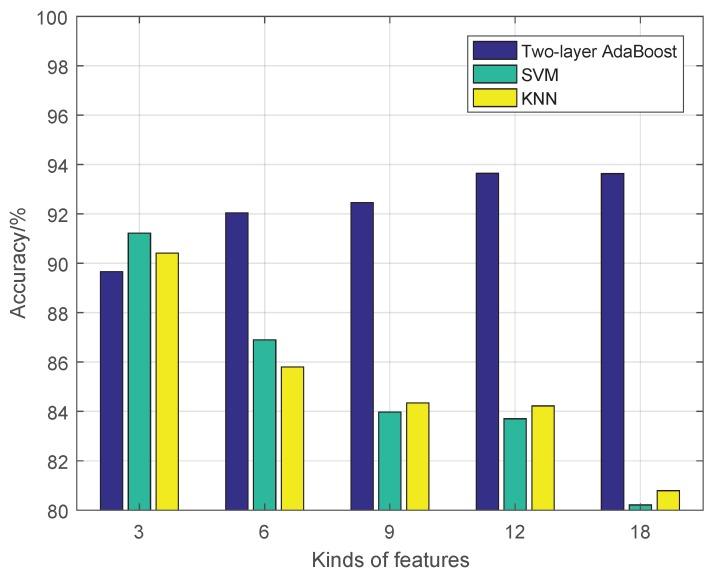
Comparison of different standard algorithms.

Figure 16 shows the comparison results of three algorithms, two-layer AdaBoost, SVM and KNN, under different number of kinds of features {Type,*D*,*C*}. SVM and KNN using CFD fusion strategy and two-layer AdaBoost performed best except for a small number kinds of features. With the increase of number of features, two-layer AdaBoost gradually improves and is obviously better. When the dimension os higher, the performance of SVM and KNN are not as good as that with a relatively low number of the dimensions, which shows that both standard algorithms are limited when facing an overfitting phenomenon. Two-layer AdaBoost overcomes the phenomenon of overfitting and improves the accuracy when faced with high-dimensional features.

We shrunk the testing samples by 1/2, 1/3, and 1/4 of formal testing dataset size and show the results in Table 6. It can be concluded that the number of testing samples does not affect the results much.

### 5.4. Comparison of Different Fusion Algorithms

To explain the superior effect of two-layer AdaBoost, we compared it with three other methods, as listed in Table 7.

**Figure 17:**
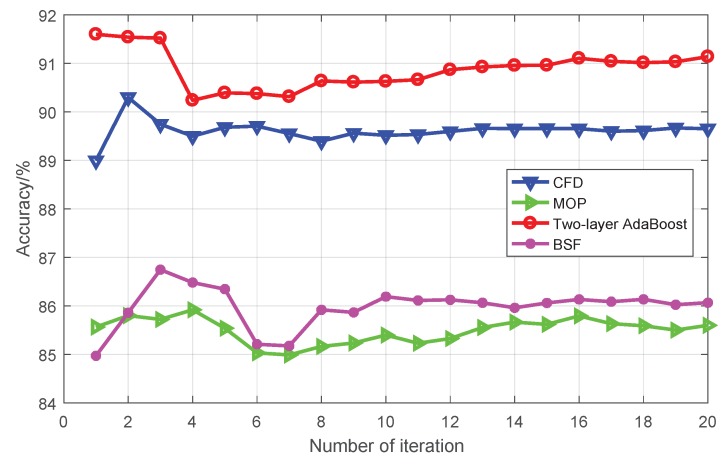
Comparison of different fusion algorithms.

The comparison results of four fusion algorithms are shown in Figure 17 where the abscissa represents the number of experiment repetitions and the ordinate denotes the average accuracy of multiple experiments. Two-layer AdaBoost performed best, followed by CFD, while MOP and BSF performed poorly. In essence, two-layer AdaBoost and CFD belong to fusion algorithms, while MOP and BSF only use one type of feature information. Therefore, multiple feature fusion algorithms work better than single feature methods.

**Figure 18:**
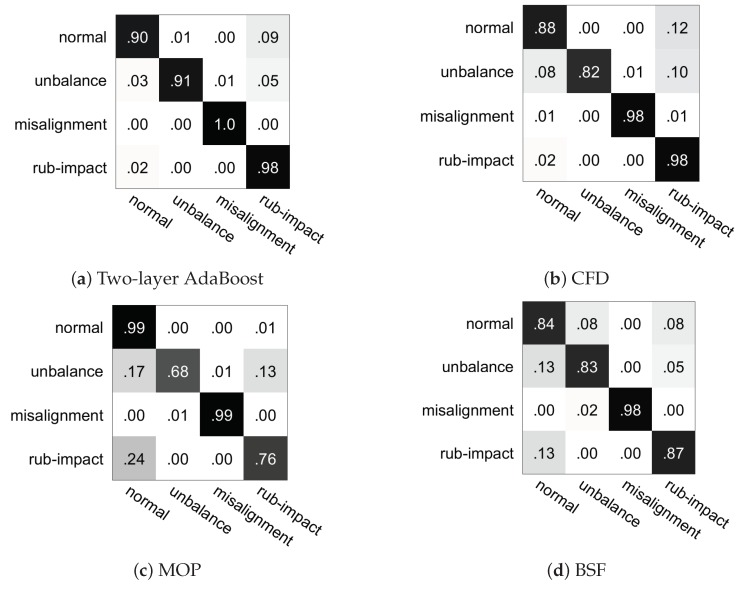
Confusion matrix of different algorithms.

Figure 18 shows the fusion matrices of the four algorithms with one iteration and reflects classification accuracy of the four classes separately. Two-layer AdaBoost classifies the four classes correctly on average and its single class accuracy ranges from 90% to 100%, and only normal samples are easily misclassified into rub-impact class. The classification precision of other three algorithms on different classes of samples is different. For example, both misalignment and rub-impact are well-classified by CFD and the recognition accuracy is up to 98%, while the precision is lower than 90% for normal and imbalance. The recognition rate of unbalance only reaches 82% and many samples are misclassified into normal and rub-impact classes. It can be concluded that two-layer AdaBoost can not only improve the overall accuracy of the dataset, but it can also effectively distinguish samples of each class averagely. It avoids the issue that some classes of samples are particularly well classified while the others are poorly distinguished.

### 5.5. Summary of the Experiments

The series of experiments proves the robustness and applicability of the vibration image and two-layer AdaBoost. However, the purpose of our algorithm is not to show our features are better than all traditional features but is to propose a new idea that is different from the traditional signal processing methods, and explore the coupling relationship between sensors at different locations. By vibration image, more advanced methods in the field of image processing and computer vision can be introduced, which are not limited to the three feature extraction methods proposed by us. In practice, faults often need to be described from multiple views. Hence, various methods including vibration image, and basic time or frequency methods can be used for multi-feature fusion, and various features complement each other to achieve a more comprehensive description of the fault.

## 6. Conclusions

In this manuscript, we propose vibration image to describe faults in rotary machinery. Vibration image combines two channels of vibration signals to discover the coupling relationship between different signals from sensors. Three features, HVI, HOVI, and 2DFFT, are designed based on vibration images in terms of signal amplitude, signal phase and frequency domain, respectively. To make full use of feature information, two-layer AdaBoost is proposed to overcome overfitting and fuse multiple features. Experiments showed that our algorithm is efficient and performs better than traditional methods.

In the future, we intend to develop more effective descriptions of vibration images to achieve better accuracy, and apply vibration images to online fault detection and fault prediction. Vibration images will also be researched to extend from 2D plane to high-dimensional space to discover coupling information among multiple channels of signals.

## Figures and Tables

**Table 1:** Dataset setting.

Classes	Number of Training Samples	Number of Testing Samples
Normal	150	260
Unbalance	150	358
Misalignment	150	172
Rub-impact	150	55

**Table 2:** The relationship between *D* and feature dimension number.

Mark D	1	2	3	4	5	6	7	8	9	10	11	12
HVI	20	30	40	50	60	70	80	90	100	120	150	200
HOVI	20	30	40	50	60	70	80	90	100	120	150	200
2DFFT	72	128	200	288	392	512	648	800	968	1152	1800	3200

**Table 3:** The relationship between *C* and feature combinations.

Mark C	1	2	3	4	5	6	7	8	9	10
Combination	X1-Y1	X1-X2	X1-Y2	X1-Z	Y1-X2	Y1-Y2	Y1-Z	X2-Y2	X2-Z	Y2-Z

**Table 4:** Best accuracy of single feature combination.

Types	HVI	HOVI	2DFFT
D	8	7	10
C	3	6	3
Accuracy	79.5%	84.4%	87.7%

**Table 5:** Features in the two feature groups.

Features	HVI-1	HOVI-1	2DFFT-1	HOVI-2
Type	HVI	HOVI	2DFFT	HOVI
C	4	9	8	4
D	1	7	7	11

**Table 6:** Results under different size of testing dataset.

Size of Testing Dataset	1	1/2	1/3	1/4
6 kinds of features	92.0%	91.0%	93.3%	90.1%
18 kinds of features	93.6%	92.2%	92.9%	92.0%

**Table 7:** Fusion algorithms for comparison.

Abbreviation	Explanation
Two-layer AdaBoost	First layer for feature dimension reduction and second layer for information fusion and classification (details seen Section 3.2)
CFD	Combine all features together directly, and use AdaBoost for training and testing
MOP	Similar to two-layer AdaBoost, omit the second layer and maximize the output of first layer (MOP). View the maximum corresponding class as the final result.
BSF	Train all types of features with AdaBoost separately and select the best performance of single feature (BSF) as the final result.
